# Enhanced Eye Velocity With Backup Saccades in vHIT Tests of a Menière Disease Patient: A Case Report

**DOI:** 10.3389/fsurg.2021.727672

**Published:** 2021-12-08

**Authors:** Maria Montserrat Soriano-Reixach, Jorge Rey-Martinez, Xabier Altuna, Ian Curthoys

**Affiliations:** ^1^Neurotology Unit, Department of Otorhinolaryngology Head and Neck Surgery, Donostia University Hospital, Donostia-San Sebastian, Spain; ^2^Biodonostia Health Research Institute, Otorhinolaryngology Area, Osakidetza Basque Health Service, San Sebastián, Spain; ^3^Vestibular Research Laboratory, School of Psychology, The University of Sydney, Sydney, NSW, Australia

**Keywords:** Meniere's disease, backup saccades, enhanced eye velocity, endolymphatic hydrops, vHIT, case report, VOR (vestibulo-ocular reflex)

## Abstract

Reduced eye velocity and overt or covert compensatory saccades during horizontal head impulse testing are the signs of reduced vestibular function. However, here we report the unusual case of a patient who had enhanced eye velocity during horizontal head impulses followed by a corrective saccade. We term this saccade a “backup saccade” because it acts to compensate for the gaze position error caused by the enhanced velocity (and enhanced VOR gain) and acts to return gaze directly to the fixation target as shown by eye position records. We distinguish backup saccades from overt or covert compensatory saccades or the anticompensatory quick eye movement (ACQEM) of Heuberger et al. (1) ACQEMs are anticompensatory in that they are in the same direction as head velocity and so, act to take gaze off the target and thus require later compensatory (overt) saccades to return gaze to the target. Neither of these responses were found in this patient. The patient here was diagnosed with unilateral definite Meniere's disease (MD) on the right and had enhanced VOR (gain of 1.17) for rightward head impulses followed by backup saccades. For leftwards head impulses eye velocity and VOR gain were in the normal range (VOR gain of 0.89). As further confirmation, testing with 1.84 Hz horizontal sinusoidal head movements in the visual-vestibular (VVOR) paradigm also showed these backup saccades for rightwards head turns but normal slow phase eye velocity responses without backup saccades for leftwards had turns. This evidence shows that backup saccades can be observed in some MD patients who show enhanced eye velocity responses during vHIT and that these backup saccades act to correct for gaze position error caused by the enhanced eye velocity during the head impulse and so have a compensatory effect on gaze stabilization.

## Introduction

Video head impulse testing of healthy subjects usually results in the eye velocity matching the head velocity, so vestibulo-ocular response (VOR) gain is about 1.0 and gaze remains on the earth fixed target. Vestibular hypofunction causes reduced eye velocity during the impulse, with corrective (compensatory) saccades—either overt or covert—to correct the gaze error and return gaze to the earth fixed target. Recently there have been reports of enhanced eye velocity (and so enhanced VOR gain) during head impulse testing of some patients with Menière's Disease (MD) (2–4). Such enhanced eye velocity could be caused by peripheral vestibular disease, such as endolymphatic hydrops related diseases or central vestibular dysfunction, such as cerebellar disease (5). Or the enhancement could be due to a recording artifact (6).

In usual vHIT testing of patients with vestibular hypofunction and reduced eye velocity during the head impulse, the corrective saccade is called compensatory since it returns the gaze to the target by canceling out the gaze position error caused by the inadequate eye velocity. In the case of enhanced eye velocity, a corrective saccade is also necessary to return gaze to the fixation target. Therefore, this corrective saccade is also compensatory in the sense that it cancels the gaze position error caused by the enhanced eye velocity. Although this saccade is compensatory, it is in the opposite direction to the common compensatory saccade recorded in patients with vestibular hypofunction. To avoid confusion we have used the term “backup saccade” to refer to this corrective saccade after enhanced eye velocity. Until now, when enhanced vestibular slow phase eye velocity during head impulses have been observed on vHIT testing, no corrective backup saccades have been described (3). In this case report we describe, for what we think is the first time, a patient with definite MD with enhanced eye velocity (and so enhanced VOR gain) during head impulse testing, who showed compensatory saccades (“backup saccades”). These backup saccades were observed on clinical HIT tests, recorded on head impulse testing using vHIT and further confirmed by their occurrence during visual-vestibular reflex (VVOR) testing.

## Case Report

A 74-year-old woman, with left eye amblyopia and no other medical history of interest, was diagnosed with unilateral definite Menière's disease (MD) of the right ear in 2016 according to the following Bárány Society diagnostic criteria for MD7: (1) Two or more spontaneous episodes of vertigo, each lasting 20 min to 12 h, (2) Low-medium frequency sensorineural hearing loss in one ear, defining the affected ear, on at least one occasion prior, (3) Fluctuating aural symptoms (hearing, tinnitus, or fullness) in the affected ear, (4) Not better accounted for by another vestibular diagnosis (7).

**Figure 1:**
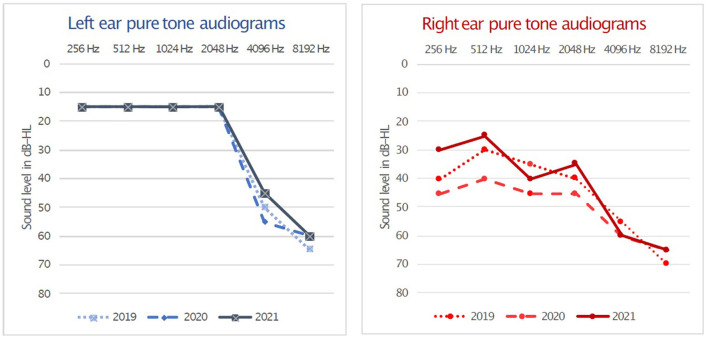
Right and left pure tone air-conduction audiograms from the last 3 years. Unilateral right fluctuating low and mid-frequencies hearing loss with a PTA 40 dB-HL is present at testing in 2019, 2020, and 2021 while normal hearing was obtained in left ear on these occasions with a PTA of 20 dB-HL. Hearing loss levels and PTA calculation methods according to BIAP 2017 (www.biap.org).

During the last 5 years she reported unilateral right fluctuating low and had mid-frequency hearing loss (Figure 1) with mild sensorineural hearing loss of the right ear (PTA 40 dB-HL). She had normal hearing of the left ear [pure tone average (PTA), 20 dB-HL according to BIAP 2017 (www.biap.org)]. During this 5-year period, the fluctuating hearing loss on the right was accompanied by recurrent vertigo attacks lasting 30–60 min with tinnitus and fullness in the right ear. These attacks occurred monthly before medical treatment. Good control of vertigo attacks was achieved with Betahistine (16 mg every 8 h) and four doses of intratympanic corticoid of Dexamethasone (1.5 ml with a dilution of 15 mg/ml) to the right ear on two occasions (2017 and 2019). In the most recent consultation, she reported occasional vertigo attacks with mild instability, which did not interfere with her daily life. The cerebral and posterior fossa magnetic resonance images showed no alterations; also, no other general or neurological symptoms were presented during the 5-year period.

**Figure 2:**
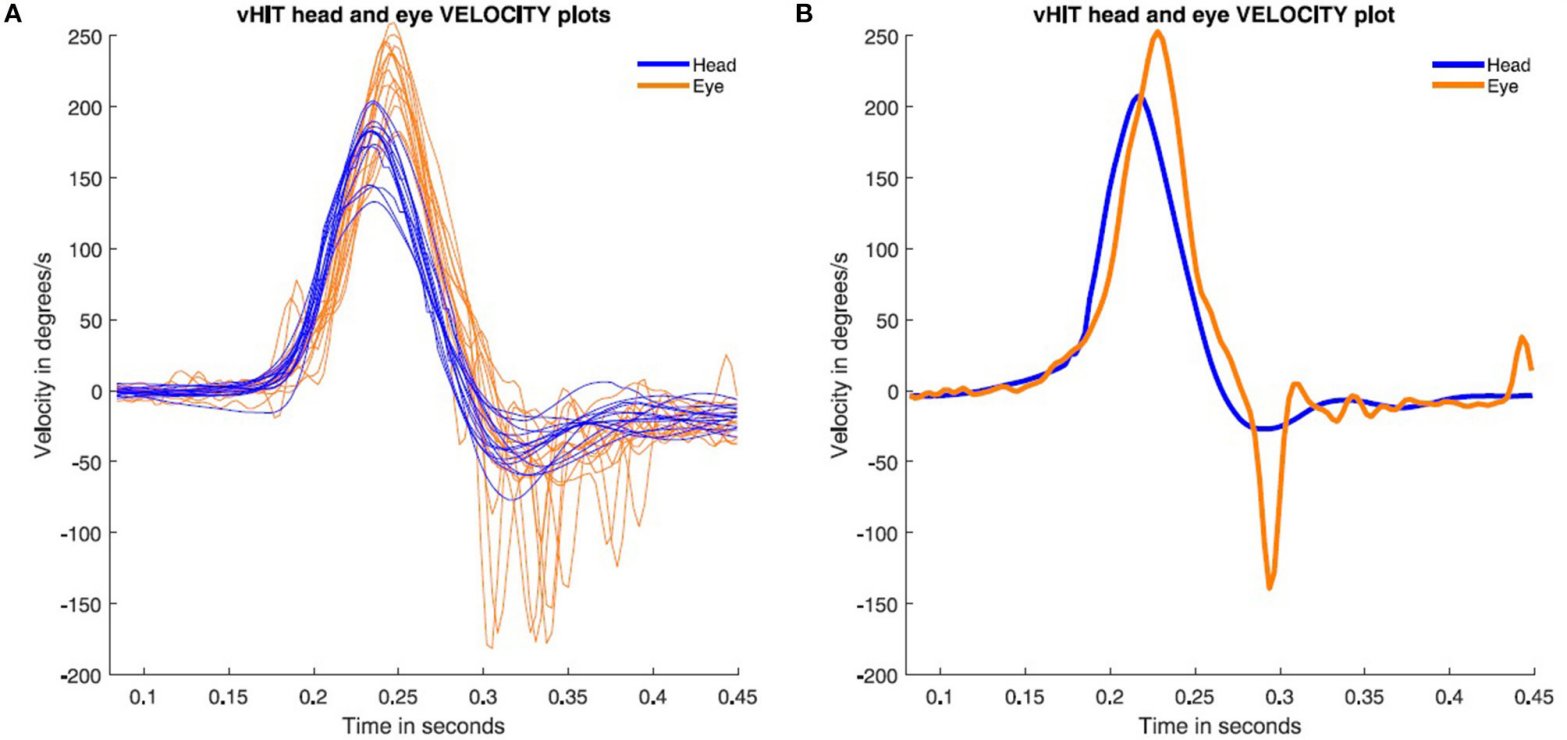
Rightward video head impulse test responses performed during the patient's most recent test (2021). The eye velocity (orange) is enhanced compared to head velocity (blue). This will result in the eye being off target and in order to correct that gaze position error (Figure 4) the eye makes a compensatory backup saccade. **(A)** All the right-side impulses are plotted with backup saccades present on all impulses. **(B)** A single rightward head impulse to show the onset of the backup saccade exactly.

On physical examination, bilateral otoscopy was normal and her smooth pursuit appeared normal at bedside testing. On horizontal head impulse testing with the instruction to fixate an earth fixed target, she showed corrective saccades when her head was turned to the affected (right) side (Supplementary Video 1), but no corrective saccades for leftwards head impulses. vHIT testing using ICS Impulse system showed exactly what was happening. During rightwards head impulses the patient had enhanced slow phase eye velocity (i.e., with eye velocity greater than head velocity, so VOR gains for the right were >1) (Figure 2, Supplementary Video 2). VOR gain was calculated using the area under the desaccaded eye velocity record divided by the area under the head velocity curve for VOR gain measurement as is standard for the ICS Impulse system. Figure 1 shows that at the end of each rightward head impulse there was a corrective saccade which acted to eliminate the gaze position error caused by the enhanced eye velocity and so this saccade is compensatory. As is clear from Figure 1, saccades rarely occurred during the rightward head turn, so desaccading was not required for VOR gain calculation. The measured VOR gain for rightward impulses was 1.17, and for leftward impulses was 0.89. As discussed above, we termed this unusual saccade a “backup saccade” to distinguish it from the usual overt or covert saccades, which act to correct for gaze position error when there is reduced slow phase eye velocity in patients with vestibular hypofunction. The backup saccade acts to correct gaze position error, with the key point being that here the gaze position error is due to vestibular hyperfunction rather than vestibular hypofunction. That result is very clearly shown by the eye position records (**Figure 4**).

**Figure 3:**
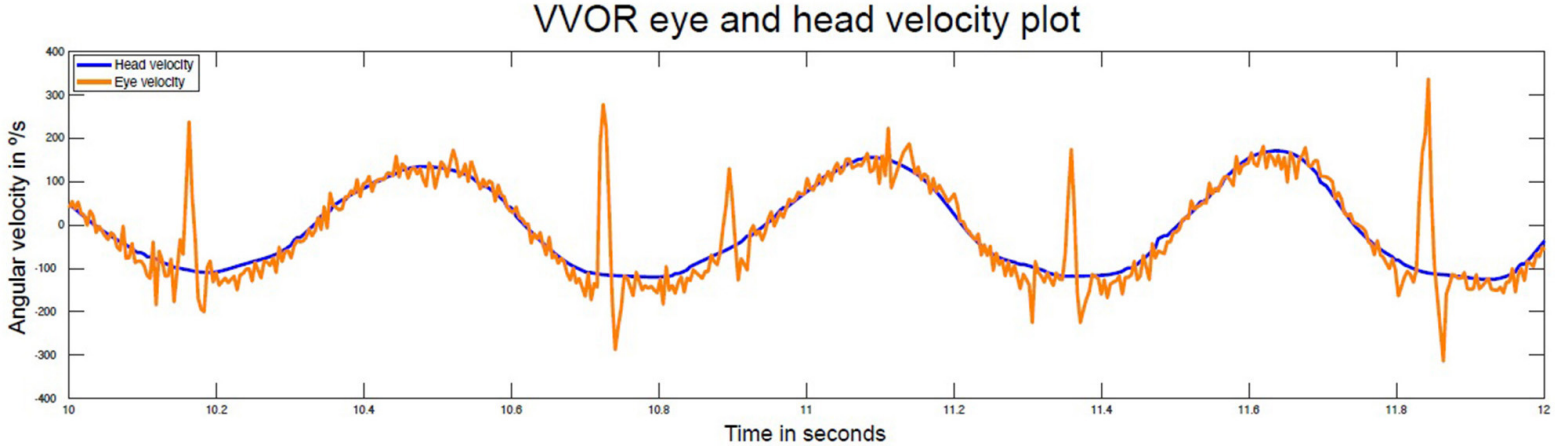
Time series of head and eye velocity during the visual vestibular-ocular (VVOR) interaction test at 1.84 Hz. There is evidence of enhanced eye velocity for rightward head turns and backup saccades are present in each rightward head turn. Normal eye velocity responses occur for leftward head rotations. In this VVOR figure, negative velocity values are assigned to right direction movement velocity. The VOR gain for the right and left sides were 1.07 and 0.91, respectively.

This patient also showed backup saccades during rightward head movements in low frequency visual vestibular (VVOR) testing (Figure 3) at a frequency of 1.84 Hz. The measured VOR gain on the right side was 1.07 and 0.91 on the left (using the gain calculation method published by Rey-Martinez et al. and Soriano-Reixach et al. (2, 8).

**Figure 4:**
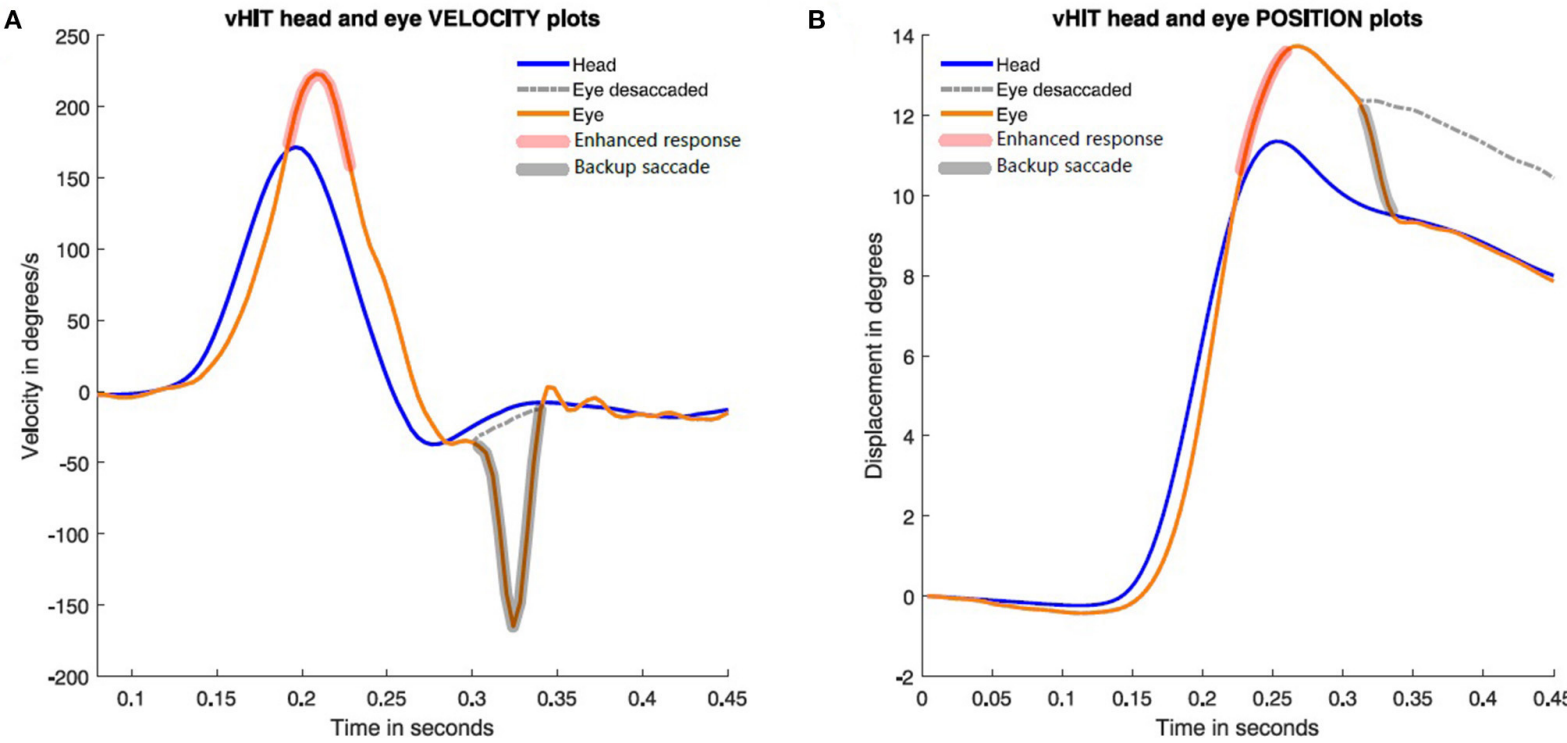
Time series plots of eye velocity and corresponding eye position during a single rightward head impulse to show how the backup saccade corrects for the gaze position error. In **(A)**, the enhanced eye velocity (red shadow brush) is followed at the end of the head impulses by a backup saccade (black shadow brush). In **(B)**, head and eye position during the impulse are plotted. The dashed line shows eye position without backup saccade.

The backup saccade we report here is distinctly different from the anticompensatory quick eye movements (ACQEM) reported by Heuberger et al. (1) during head impulse testing of some patients with Meniere's Disease for the following reasons;

ACQEMs occur during the head impulse and take the gaze off the target, in the direction of the head velocity and so are anticompensatory in the sense that the ACQEM will take gaze off the fixation target and so require a corrective saccade at the end of the head impulse to return gaze to the earth fixed target. These corrective saccades after ACQEMs are clearly shown in Heuberger Figure 1. In contrast, backup saccades occur at the end of the head impulse and return gaze to the target directly and so are compensatory. They act to correct the gaze position error entailed by enhanced eye velocity during the head impulse (Figures 2, 4).
The latency for the ACQEM is consistently around 96 ms from the onset of the head impulse by Heuberger et al. (1), whereas the backup saccade has a longer variable latency of around 150–200 ms (see Figure 1).

We also note that the backup saccade is distinctly different from the very early compensatory “saccade-like” response reported by Curthoys et al., in some MD patients with enhanced eye velocity (3). The latency of these “saccade-like” responses is very short and much shorter than ACQEMs and backup saccades, as well as being in the opposite direction to ACQEMs. In the Curthoys et al. study, it was suggested that the enhanced eye velocity might be due to hydrops, in accord with the effect of glycerol and modeling and imaging data (9). However, another possibility is that cerebellar dysfunction could result in enhanced VOR gain (5). Alternatively the enhanced eye velocity may be due to a calibration error (6). That is unlikely because it would affect both leftward and rightward eye velocity responses, whereas backup saccades were only found on rightward head impulses.

In the clinical series published by Vargas et al. it was reported that of 56 patients with MD, 39.6% of them had enhanced VOR responses on vHIT testing, but in most of these cases with enhanced VOR gain none presented backup saccades as reported here. Only two cases in that clinical series (one of them being the case presented here), had evident backup saccades (i.e., only 8.6% of the MD patients with enhanced VOR gain on vHIT). Why are backup saccades not more commonly seen in patients with enhanced eye velocity? It has been suggested that this could be due to the very different eye velocity trajectories during the increasing head velocity and decreasing head velocity phases of the head impulse [Curthoys et al. (3); Figure 1]. It may be that the eye position error during the acceleration phase may be partially corrected by the eye position error during the deceleration phase, resulting in such a small final eye position error that it is not necessary for any correction.

The main limitation of this case report is the possible effect of left eye amblyopia on right eye recorded vHIT responses. At this moment, we did not find any bibliographic reference about the possible effect of contralateral amblyopia on vHIT testing, but this and many of the previous discussed questions should be investigated on further research as other possible cause associated with the reversed backup saccades.

A recent report confirming the presence of very early saccade responses during active head impulses has highlighted the future need for detailed examination of these various saccades or saccade-like responses during head impulse testing, including consideration of a factor which has been shown to affect saccades—the patient's age (10, 11).

## Conclusion

Backup saccades can be observed in some MD patients with enhanced eye vHIT responses. These backup saccades are compensatory but are in the opposite direction to the usual compensatory saccades recorded in patients with reduced vestibular function.

## Data Availability Statement

The data in this article is not readily available due to consent needed to use patient data. Reasonable requests can be made to the corresponding author/s.

## Ethics Statement

The studies involving human participants were reviewed and approved by Donostia University Hospital. The patients/participants provided their written informed consent to participate in this study. Written informed consent was obtained from the individual(s) for the publication of any potentially identifiable images or data included in this article.

## Author Contributions

MS-R and JR-M contributed to conception and design of the study and organized the database. JR-M performed the statistical analysis and wrote sections of the manuscript. MS-R wrote the first draft of the manuscript. All authors contributed to manuscript revision, read, and approved the submitted version.

## Conflict of Interest

The authors declare that the research was conducted in the absence of any commercial or financial relationships that could be construed as a potential conflict of interest.

## Publisher's Note

All claims expressed in this article are solely those of the authors and do not necessarily represent those of their affiliated organizations, or those of the publisher, the editors and the reviewers. Any product that may be evaluated in this article, or claim that may be made by its manufacturer, is not guaranteed or endorsed by the publisher.

## Supplementary Material

Supplementary Video 1 Examples of backup saccades during rightward head impulses.

Supplementary Video 2 External video of vHIT testing. Backup saccades are evident in the absence of observable recording artifacts such as google slippage.
